# Increased Production of Pathogenic, Airborne Fungal Spores upon Exposure of a Soil Mycobiota to Chlorinated Aromatic Hydrocarbon Pollutants

**DOI:** 10.1128/spectrum.00667-23

**Published:** 2023-06-07

**Authors:** Celso Martins, Daryna Piontkivska, Dalila Mil-Homens, Paula Guedes, João M. P. Jorge, João Brinco, Cátia Bárria, Ariana C. F. Santos, Ricardo Barras, Cecília Arraiano, Arsénio Fialho, Gustavo H. Goldman, Cristina Silva Pereira

**Affiliations:** a Instituto de Tecnologia Química e Biológica António Xavier, Universidade Nova de Lisboa, Oeiras, Portugal; b Institute for Bioengineering and Biosciences and Institute for Health and Bioeconomy, Instituto Superior Técnico, University of Lisbon, Lisbon, Portugal; c Department of Bioengineering, Instituto Superior Técnico, University of Lisbon, Lisbon, Portugal; d CENSE (Center for Environmental and Sustainability Research)/CHANGE (Global Change and Sustainability Institute), NOVA School of Science and Technology, NOVA University Lisbon, Caparica, Portugal; e Faculdade de Ciências Farmacêuticas de Ribeirão Preto, Universidade de São Paulo, Ribeirão Preto, Brazil; Geisel School of Medicine at Dartmouth

**Keywords:** *Aspergillus fumigatus*, *Galleria mellonella* infection model, organic pollution, soil mycobiota, fungal virulence

## Abstract

Organic pollutants are omnipresent and can penetrate all environmental niches. We evaluated the hypothesis that short-term (acute) exposure to aromatic hydrocarbon pollutants could increase the potential for fungal virulence. Specifically, we analyzed whether pentachlorophenol and triclosan pollution results in the production of airborne fungal spores with greater virulence than those derived from an unpolluted (Control) condition. Each pollutant altered the composition of the community of airborne spores compared to the control, favoring an increase in strains with *in vivo* infection capacity (the wax moth Galleria mellonella was used as an infection model). Fungi subsisting inside larvae at 72 h postinjection with airborne spore inocula collected in polluted and unpolluted conditions exhibited comparable diversity (mainly within Aspergillus fumigatus). Several virulent Aspergillus strains were isolated from larvae infected with the airborne spores produced in a polluted environment. Meanwhile, strains isolated from larvae injected with spores from the control, including one A. fumigatus strain, showed no virulence. Potential pathogenicity increased when two Aspergillus virulent strains were assembled, suggesting the existence of synergisms that impact pathogenicity. None of the observed taxonomic or functional traits could separate the virulent from the avirulent strains. Our study emphasizes pollution stress as a possible driver of phenotypic adaptations that increase Aspergillus pathogenicity, as well as the need to better understand the interplay between pollution and fungal virulence.

**IMPORTANCE** Fungi colonizing soil and organic pollutants often meet. The consequences of this encounter constitute an outstanding question. We scrutinized the potential for virulence of airborne fungal spores produced under unpolluted and polluted scenarios. The airborne spores showed increased diversity of strains with higher infection capacity in Galleria mellonella whenever pollution is present. Inside the larvae injected with either airborne spore community, the surviving fungi demonstrated a similar diversity, mainly within Aspergillus fumigatus. However, the isolated Aspergillus strains greatly differ since virulence was only observed for those associated with a polluted environment. The interplay between pollution and fungal virulence still hides many unresolved questions, but the encounter is costly: pollution stress promotes phenotypic adaptations that may increase Aspergillus pathogenicity.

## INTRODUCTION

Pollution is among the leading causes of disease and premature death (1), and yet the impacts of pollution on public health are generally ignored (1, 2). Pollution is intimately linked with climate change since it leads to major geological/geochemical or atmospheric alterations that negatively impact human lives (e.g., global warming) (2) with associated impacts that transfer to the economy (3). There is growing concern about the impacts of pollution on human health and the need to better understand the associated risks (4). One unseen consequence of the environmental changes caused by pollution is the emergence of novel microbial pathogens (5, 6): a complex multifactorial phenomenon influenced by various genetic adaptations, ecological drivers, and selective pressures imposed by the human host (6, 7). Understanding this phenomenon has major public health and economic implications. For example, pollution can alter the skin microbiota (8) and hence the epidemiology and severity of cutaneous disorders (9). Pollution also contributes to the spread of antimicrobial resistance genes in some pathogenic bacteria (6), although a connection to microbial infection is yet unproven. Peaks of air pollution have also been correlated with waves of the SARS-CoV-2 pandemic (10). The increased burden of fungal infections in humans and the emergence of clinical azole resistance and multidrug-resistant fungi (e.g., Aspergillus fumigatus and Candida auris) have been linked to the application of fungicides in agriculture (11).

Fungi are ubiquitous; they are found in the stratosphere, deserts, deep ocean sediments, Antarctic glaciers, and even in the human gut (12). There are nearly 150,000 known fungal species (2 to 3.8 million are estimated to exist [13]), including hundreds that can cause disease in humans (12, 14). Fungal pathogens receive much less attention than their bacterial and viral counterparts, despite the staggering global burdens of pre- and postharvest contamination of agricultural products and of fungal disease—from superficial, mucosal, and allergic infections to chronic severe and acute invasive infections (14–17). The latter are responsible for more than 1.6 million deaths annually, affecting mainly vulnerable, immunocompromised patients of all ages. Human exposure to airborne fungal spores (and mycelia/hypha fragments as well) is universal; when inhaled, fungal spores cause adverse health effects (18). Different mycobiomes are associated with lung and skin cancers where inhaled/deposited fungal spores are prevalent (19). Many clinically relevant fungal pathogens, including A. fumigatus and Candida albicans, can be found in the environment, where environmental pressures not only contribute to the expansion of their ecological range and their long-distance dispersal but also affect the evolution of novel traits, including virulence and antifungal resistance (17, 20).

More details are needed of the interplay between ecology and fungal virulence and pathogenicity in the context of pollution. Previously, we observed that the efficient degradation of pentachlorophenol (PCP) by a metacommunity of fungi resulted in potentially pathogenic tradeoffs, specifically, the dysregulation of carbon and nitrogen metabolisms, the secretion of proteins associated with pathogenesis, and a major decrease in overall susceptibility to a common fungicide (21). The present study outlines a further proof of concept that pollution critically alters the structure of fungal communities and favors the dispersal of airborne spores with a higher degree of pathogenicity than those derived from an unpolluted source.

We designed and fabricated a custom cultivation system to simulate acute exposure to PCP and triclosan (TCS) and to collect the resulting airborne spores (allowing for the systematization of serial tests). The chosen pollutants have a long history of use and persist in virtually all ecosystems (22, 23). Both pollutants altered the composition of airborne spores, favoring the rise of Aspergillus strains with greater virulence toward the wax moth Galleria mellonella with an infection capacity similar to that of clinical fungal isolates. The structure of the fungal community upon exposure to either pollutant, including key traits of the cultivable strains found inside larvae, is discussed below in detail.

## RESULTS AND DISCUSSION

### Most airborne spores derive from aspergilla.

**FIG 1:**
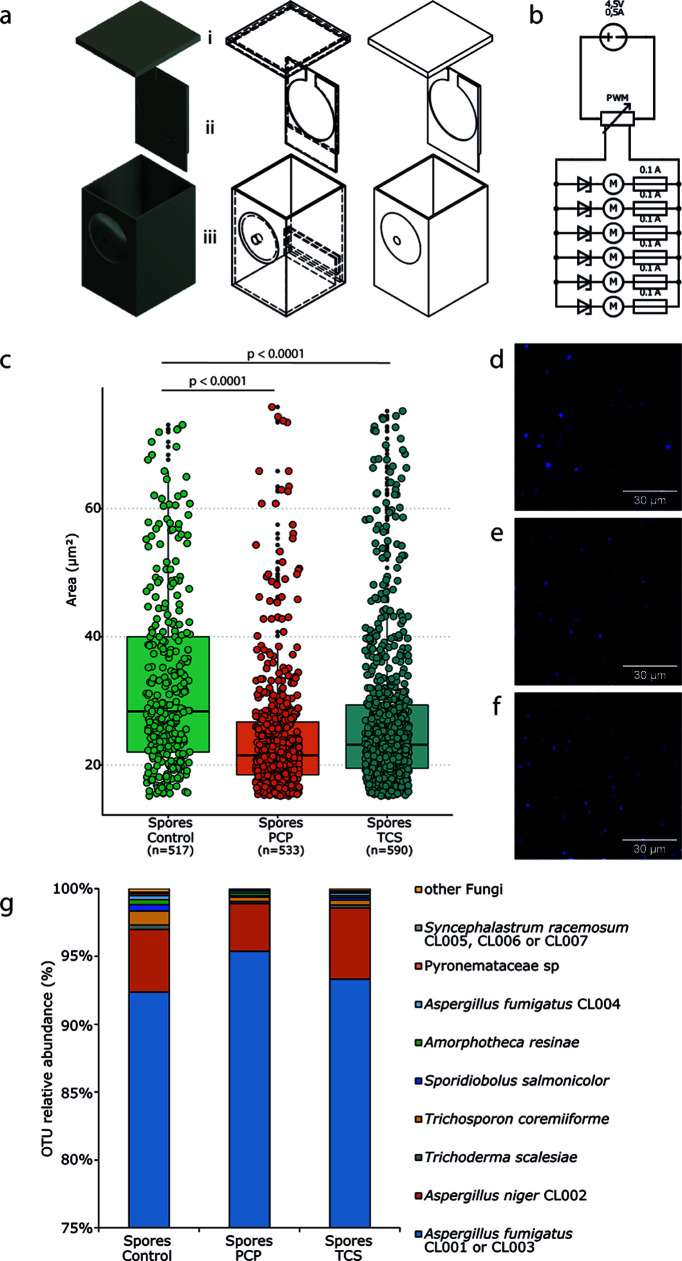
Overview of the MC3000 system and characterization of airborne fungal communities. (a and b) Schematic view of the MC3000 cultivation system (a) and the electronic circuit that operates fans for airflow (b). In greater detail, the MC3000 system is composed of (i) the upper lid, (ii) the detachable adapter to accommodate membrane filters for airborne spores’ collection, and (iii) the cultivation box (dimensioned for 50 mL of culture medium) with a hole in the front panel where an electric vane is connected to ensure airflow. (c) Boxplots show the sizes (area in μm^2^) of airborne spores collected using MC3000. (d to f) Representative micrographs show spores stained with calcofluor-white: control (d), PCP (e), and TCS (f) conditions (scale bar, 30 μm). (g) The taxonomic composition of the airborne fungal communities collected at each condition was assessed by amplicon sequencing and is represented using stacked histograms.

In the present study, we focus our attention on PCP and TCS to study how pollution influences the overall pathogenic potential of environmental fungi. TCS can be biotically converted to dichlorophenol, which is then metabolized through the hydroquinone pathway (24, 25), similar to that which occurs during the fungal degradation of PCP (26). PCP is classified as a persistent organic pollutant and its usage/production is restricted in many countries (22). TCS is classified as a contaminant of emergent concern and is one of the most commonly used antimicrobial agents, especially in biocidal, human hygiene products, including toothpaste (23). Perhaps unsurprisingly, TCS has been frequently linked to multidrug resistance in bacteria (27). The soil mycobiota grown in the MC3000 cultivation system (Fig. 1a) were able to efficiently degrade both PCP and TCS, achieving degradation levels of 70.8 ± 3.6% and 94.9 ± 2.2%, respectively, after 10 days (see Table S1 and Fig. S1 in the supplemental material). A similar degree of PCP degradation was observed for this soil mycobiota using a multiwell cultivation approach (21). The gelling agent added in the broth medium did not affect PCP degradation. TCS degradation was notably high, underlining the superior capacity of a soil mycobiota to degrade pollutants (21, 26).

The yields of airborne fungal spores collected, i.e., adhered to the MC3000 membranes, in cultures exposed to PCP and TCS were comparable: 3.4 × 10^+07^ and 3.9 × 10^+07^, respectively. Under unpolluted (control) conditions, a smaller number of spores was collected, but within the same order of magnitude (1.5 × 10^+07^). Under either condition, our customized cultivation system (Fig. 1a and b) allowed for the recovery of a high number of spores; appropriate amounts with which to test hypotheses and perform complementary and validation experiments. Heterogeneity of spore size (area, expressed in μm^2^) was observed under all conditions (Fig. 1c; see also Table S2). The average sizes were 24.54 ± 9.87 μm^2^ (*P* ≤ 0.001) and 26.87 ± 9.30 μm^2^ (*P* ≤ 0.001) for the PCP and TCS conditions and 32.49 ± 13.79 μm^2^ for the control conditions. The higher average size of spores from unpolluted conditions is apparent in the corresponding micrographs (Fig. 1d to f). Preliminary tests showed similar spore sizes when comparing airborne spores to those deposited onto solid media (see Fig. S2), supporting the idea that the size of airborne spores is representative of each mycobiota.

Amplicon short-read sequencing was used to characterize the taxonomic composition of the subpopulation of each airborne spore. A total of 464,293 reads matched the fungal internal transcribed spacer (ITS) region, corresponding to the identity of 184 operational taxonomic units (OTUs) under the control condition, 152 OTUs under PCP conditions, and 184 OTUs under TCS conditions (see Data Set S1). The OTUs were grouped into 33 species clusters according to their NCBI accession numbers (see Table S3). Using read BLAST, the OTU diversity of the airborne spores’ mycobiota was compared to that obtained when the same inoculum was grown using a metacommunity approach (21) (see Data Set S1). All of the OTUs identified were present in the inoculum (pregrowth), and 93% were present in the full-grown metacommunity (21). This result confirms that using the MC3000 cultivation system did not alter the mycobiota composition. Of the 17 OTUs previously identified as significant PCP assimilators (21), 14 were present in the airborne spores collected, irrespective of different relative abundances. This deviation can derive from differences in the spore production rates and the influence of the cultivation methodology used. Overall, the amplicon sequencing data supported our strategy of steadily increasing the complexity of lab simulations to better understand the dynamics of the mycobiota composition. It is important to perform these studies prior to carrying out *in vivo* infection studies.

The taxonomic diversity of the airborne spores under all conditions is clearly dominated by Aspergillus spp., predominantly A. fumigatus (>90%), distantly followed by Aspergillus niger (~3% under PCP conditions and ~5% under unpolluted and the TCS conditions; Fig. 1g). The dominance of Aspergillus spp. is consistent with their widespread environmental distribution and preferential colonization of decaying organic matter. In particular, A. fumigatus has high thermotolerance (required to survive in compost piles) and is tolerant to a wide range of stresses (e.g., osmotic/oxidative stress and starvation) that are associated with life in the soil (20). This fungus is the major causative agent of respiratory and systemic fungal infections, followed by A. flavus and A. niger (28). Other fungal taxa make up at least 0.2% of the total abundance under all conditions; the following were found: *Trichoderma scalesiae*, *Amorphotheca resinae*, *Pyronemataceae* sp., *Trichosporon coremiiforme*, *Sporidiobolus salmonicolor*, and *Syncephalastrum racemosum*. All of these fungi are environmentally widespread, and only a few have been described as potential human pathogens, often as rare occurrences and with unclear epidemiology (29–31).

### Virulent airborne spores are produced when exposed to pollution.

**FIG 2:**
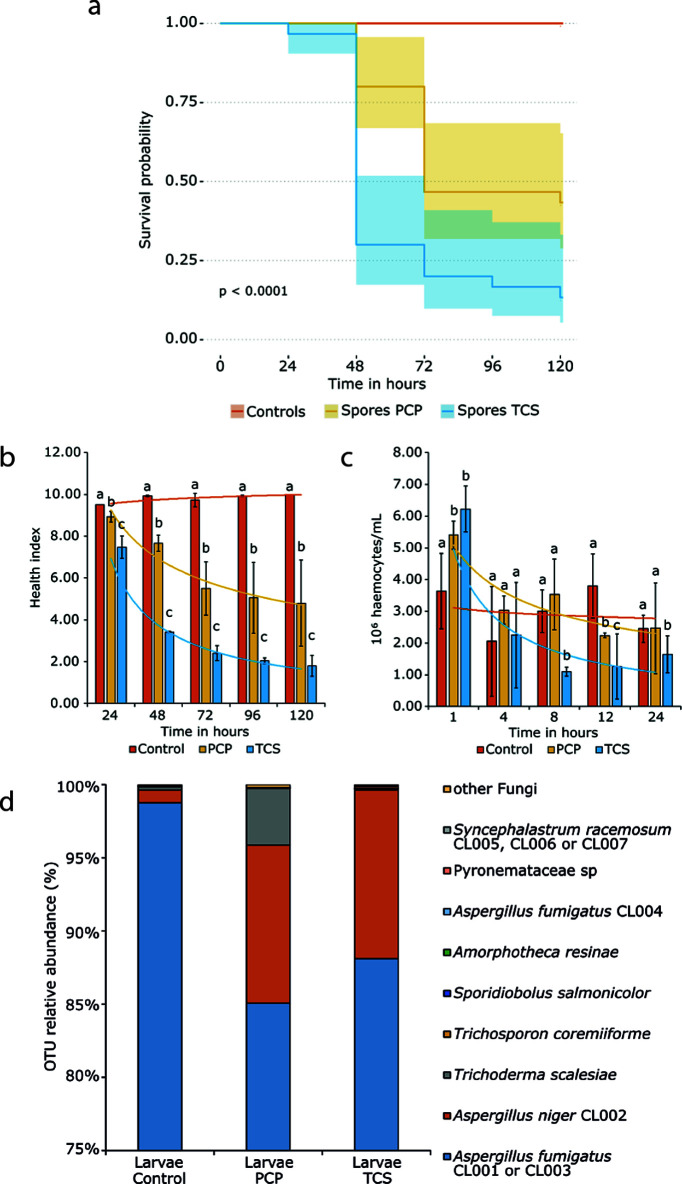
Infection studies of the airborne fungal communities and composition of the fungal community subsisting inside Galleria mellonella larvae. (a) The survival probability of Galleria mellonella larvae upon infection with 10^6^ airborne spores (per larvae) collected under each condition (unpolluted [control], PCP, and TCS) is shown using Kaplan-Meier curves. The control line overlaps the results obtained for the control (10^6^ airborne spores produced under unpolluted conditions); the injection of a saline solution (blank) and the injection of extracts from the airborne spores produced under each condition (see Data Set S2 in the supplemental material). The *P* value was computed using the log rank test embedded in the R package survminer. (b) Health indexes measured throughout the infection experiments. (c) Number of hemocytes measured during the first 24 h of infection. Significant differences between conditions (*P* < 0.05) at each time point were assessed using a Student *t* test (values are denoted using different letters), and power trend lines show data trajectory. (d) The taxonomic composition of the fungal communities proliferating inside the infected larvae was assessed by amplicon sequencing and is represented using stacked histograms.

We then assessed the survival rate of G. mellonella larvae, injected with airborne spores, as a preliminary indicator of the virulence of each subpopulation. This is a well-established model to evaluate the virulence of microbial pathogens (32, 33), specifically fungal pathogens (34), and shows a good correlation with murine models (35). The insect innate immune response is similar to that of vertebrates; hemocytes function in a similar manner to phagocytes (36, 37). Changes in hemocyte populations reveal the immune responses to fungal infections (35). An inocula of 10^6^ airborne spores from the PCP or the TCS conditions (here referred to as PCP or TCS inoculum, respectively, or collectively as test inoculum) led to low larval survival rates (Fig. 2a). This was in contrast to the unpolluted condition (here referred to as unpolluted inoculum) that resulted no death throughout the infection experiment (120 h). As a preliminary test, we pooled the airborne and deposited spores and performed infection tests using 10^7^ spores. At 24 h postinfection, almost all the larvae infected with either test inoculum died (see Table S4). This result, together with the comparable sizes of airborne and deposited spores (see Fig. S2), confirms the suitability of the airborne spore subpopulation to monitor potential risks of exposure through inhalation.

The larvae infected with the TCS inoculum displayed lower survival rates than those infected with the PCP inoculum (10^6^ airborne spores per larvae). At 48 h postinfection, survival probabilities were <30% (TCS) and >75% (PCP), and at 120 h postinfection were ~10 and ~40%, respectively (Fig. 2a). Importantly, larval death cannot be attributed to pollutants or their degradation products adsorbed to the spores. This is because the extracts, obtained from the airborne spores produced under either test condition, when injected in larvae resulted in no death (see controls in Fig. 2a; see also Data Set S2 for further details). Moreover, these extracts did not contain residual pollutants above levels that could be detected by chromatography or mass spectrometry (see Fig. S3 and S4). These results clearly show that the low survival rates of larvae infected with either test inoculum were due to the mycobiota and were not caused by any chemical contamination.

The health indexes measured throughout the infection experiments are consistent with the observed survival rates (Fig. 2b). Larvae inoculated with either test inoculum (10^6^ airborne spores per larvae) showed poor indexes of health, namely, high melanization, low mobility, no cocoon formation, and high rates of death. This is in contrast with those infected with the unpolluted inoculum that scored at the maximum for indexes of health. At 1 h postinfection, the production of hemocytes in larvae inoculated with either test inoculum increased greatly compared to the unpolluted inoculum that exhibited stable production until 24 h postinfection (Fig. 2c). This result is consistent with the fast melanization of the larvae that was observed upon inoculation (within a few minutes). Analysis of the expression of G. mellonella antimicrobial peptides encoding genes were not conclusive (see Fig. S5). Hemocyte production is used as an indicator for the action of hemocytes when the insect is attacked by infectious agents (36, 37). For prolonged postinfection periods, and for either test inoculum, the production of hemocytes progressively decreased to reach values below those found in the unpolluted inoculum at 12 h (trend lines in the bar plot; Fig. 2c). This suggests that either test inoculum could, to some extent, evade the innate immune system of the larvae, proliferating inside their bodies and consistent with previous reports (36).

### Determining the key, virulent fungi.

To determine the identities of the most abundant fungi that subsisted inside the larvae, we extracted bulk DNA from the larvae that survived until 72 h postinfection. Selecting a larval premortem stage (as opposed to collecting samples from larvae killed by fungi) allows for the standardization of conditions between test inoculum and control inoculum. Furthermore, the postmortem fungal diversity could be biased toward minor fungi that achieved optimum growth conditions once the larvae are dead. Consistent with the inoculum (Fig. 1g), the dominance of Aspergillus spp. is evident under all conditions (Fig. 2d). The relative abundance of A. niger increased under both test conditions, while the less abundant taxa became even more scarce (except for *T. scalesiae* in the PCP inoculum). Incubation temperatures were 30°C for the MC3000 experiments and 37°C for the infection experiments. As such, it simulates a real-world situation: inhaled airborne spores are subjected to higher temperatures in the animal host compared to the temperature of the environmental niche from where they originated. Such temperature differences can partially explain reductions in the relative abundance of specific fungal taxa.

**FIG 3:**
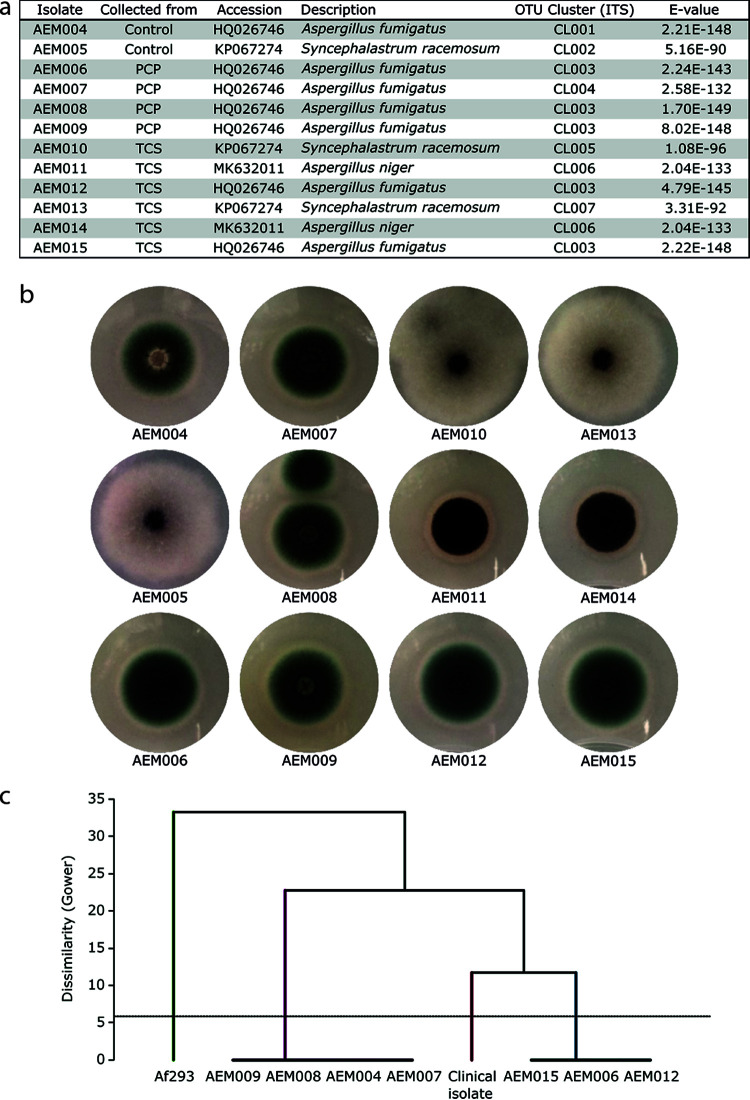
Strains isolated from infected Galleria mellonella larvae. (a) The strains isolated from larvae infected with airborne spores are listed. Internal strain accession codes, the condition from which the infecting spores were collected, the NCBI best hit accession number, the taxa, the cluster based on the alignment of the sequenced ITS regions (for amplicon sequences, see Data Set S3 in the supplemental material), and the E value obtained from BLAST analysis are provided. (b) Morphology of each strain when cultivated in potato dextrose agar. (c) Hierarchical cluster analysis based on Gower dissimilarity index shows the existence of two different lineages among the Aspergillus fumigatus strains isolated from larvae, analyzed by microsatellite genotyping (for full data, see Table S5 in the supplemental material). The laboratory model strain Af293 and a clinical isolate (AF18) were also analyzed and used for comparison.

The amplicon sequencing data revealed the major shifts that occur in the composition of the fungal community once the inoculum is transferred inside the larvae. Meanwhile, the identity of the most critical players, which cause progression of infection and ultimately death, remains cryptic. To address this question, we isolated fungal strains that endured within larvae (72 h postinfection). Consistent with the amplicon sequencing data, we isolated mostly Aspergillus spp., specifically seven A. fumigatus strains (AEM004, AEM006 to AEM009, and AEM012 to AEM015 from larvae infected with the unpolluted inoculum, the PCP inoculum, and the TCS inoculum, respectively) and two A. niger strains (both from the TCS inoculum, AEM011 and AEM014; Fig. 3a and b). Three *S. racemosum* strains (AEM005, AEM010, and AEM013) were also isolated irrespective of the fact that this species was not among the most abundant in the amplicon sequencing data (Fig. 2d). This fungus is a potential human pathogen, even if well-documented cases are rare (30). The sequences of the ITS2 region of each isolate (see Data Set S3) were matched with the amplicon sequencing data to place them within the context of the overall fungal community. However, since redundancy exists in short-read sequencing data, it is not possible to precisely distinguish different lineages within the isolated strains (Fig. 3a). Therefore, because A. fumigatus is the most notable invasive human pathogen (14, 17), we used microsatellite genotyping to achieve a higher discriminatory power within this species (38). A reference laboratory strain (Af293) and a clinical isolate (AF18) were analyzed in parallel. The results show that all A. fumigatus strains isolated from larvae cluster in two distinct lineages (Fig. 3c; see also Table S5). The clinical isolate is closely related to the lineage of the isolated strains AEM006, AEM012, and AEM015, whereas the laboratory strain is the most distant from all tested strains.

We also analyzed important phenotypic features of each of the 12 isolates, namely, the 50 effective concentration (EC_50_) of both pollutants and the minimal inhibitory concentrations (MICs) of two antifungal drugs: amphotericin B and posaconazole (see Table S6). The calculated EC_50_ values range from 4.4 to 10.5 mg L^−1^ for PCP and from 4.3 to 36.9 mg L^−1^ for TCS and did not show any correlation with the origins of the strains. For example, the A. fumigatus strain isolated from larvae infected with the unpolluted inoculum (AEM004) showed the lowest susceptibility to either pollutant, whereas the clinical isolate ranks at an intermediate position for PCP (8.1 mg L^−1^) and at the bottom for TCS (4.3 mg L^−1^). The determined MICs show also great variability, ranging from 1 to 128 mg L^−1^ for amphotericin B and from 0.06 to 4 mg L^−1^ for posaconazole. All A. fumigatus isolates are less susceptible to amphotericin B compared to either the clinical isolate or the remaining isolates but are much more susceptible to posaconazole (0.06 to 0.25 mg L^−1^) compared to the two *S. racemosum* strains (2 and 4 mg L^−1^).

**FIG 4:**
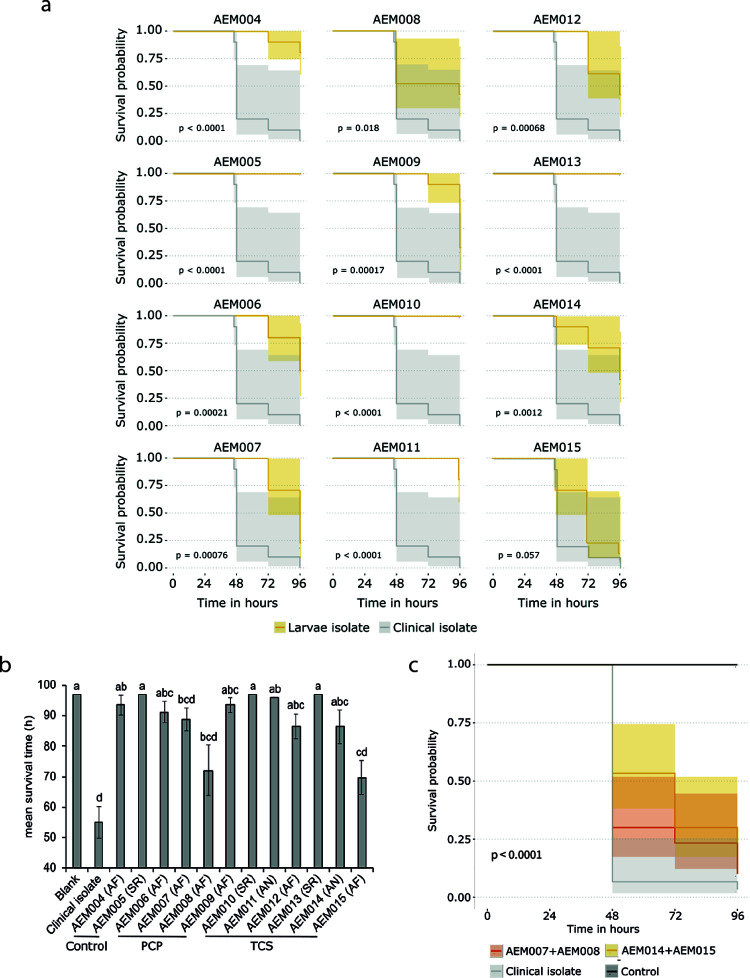
Infection capacity of each strain isolated from Galleria mellonella larvae and of artificial consortia. The infection capacity of each strain isolated from G. mellonella (survival curves in yellow) larvae and of a clinical isolate (AF18, survival curves in gray) were assessed individually (infection with 10^7^ airborne spores per larvae). (a and b) The Kaplan-Meier survival curves (a) and bar plots discriminating the mean survival times (b) for each strain reveal distinct infection capacities within *Aspergillus* species (Aspergillus fumigatus and A. niger). Significant differences (*P* < 0.05) were assessed by computing Monte Carlo simulations, followed by a Kruskal-Wallis test (see Data Set S4) and are denoted by using different letters (i.e., samples labeled with more than one letter are not different from others containing either one of the letters). (c) The infection capacity of consortia consisting of the two most virulent strains isolated from each test condition and the comparison with a clinical isolate (AF18) is shown using Kaplan-Meier survival curves (infection with 10^7^ airborne spores per larvae). The *P* values computed using the log rank test embedded in the R package survminer are displayed for each survival curve.

We observed that the *in vivo* infection capabilities of the spores (10^7^ spores injected) produced by each of the 12 strains (plus the clinical strain) were very distinct (Fig. 4a and b). These results reflect the characteristic *in vivo* infection capacity of each strain because potential transient effects, arising from exposure to either pollutant, would have been diluted by the time of testing (throughout the strain isolation procedure [i.e., several subculturings] and during the collection of spores [always obtained from new cultures]). None of the *S. racemosum* strains kill any larvae, regardless of their provenance. The remaining Aspergillus strains displayed highly variable killing capacities (i.e., low mean survival time, Fig. 4b). Among the seven A. fumigatus strains, AEM004 (unpolluted inoculum), AEM006/7 (PCP inoculum), and AEM009/12 (TCS inoculum) were benign to moderately virulent in contrast to AEM008 (PCP inoculum) and AEM015 (TCS inoculum), which were both highly virulent. The last two strains (same taxonomic cluster) are found in distinct lineages (Fig. 3c). Several studies have shown that the potential virulence of A. fumigatus strains is highly variable (39, 40). Finally, the two A. niger strains (TCS inoculum), which are placed in the same taxonomic cluster, showed contrasting *in vivo* infection capacity; AEM011 was rather benign, while AEM014 was highly virulent (Fig. 4b; see Data Set S4).

The killing efficiency of either test inoculum (see Table S4) was meaningfully higher than that of any axenic spore preparation when 10^7^ spores were injected. The mycobiota found inside the infected larvae (Fig. 2d), indicates that the most abundant strains were likely isolated. Previous studies showed that mixed populations of fungi are often found in the lungs of patients with invasive aspergillosis (41). These observations support the hypothesis that synergisms between the individual strains, that make up the consortia of either of test inoculum, increases the overall *in vivo* infection capacity. This hypothesis is consistent with the observation that mixtures of spores from two virulent strains resulted in higher killing rates compared to those caused by the axenic spores (Fig. 4c). Specifically, larvae infected with a mixture of 10^7^ spores of two strains derived from the PCP-inoculums (1:1, AEM007:AEM008) exhibited survival probabilities of close to 25% at 48 h postinfection, which decreased to almost zero at 96 h postinfection. For the TCS-inoculum (1:10, AEM014:AEM015; 10^7^ spores), survival probability was ~50% at 48 h postinfection and steadily decreased to <20% at 96 h postinfection. This result raises interesting questions about the interactions that occur between distinct strains inside an animal host. It is known that different strains produce a wider array of secondary metabolites (some of which are potentially involved in infection processes [42]) when cocultivated under laboratory conditions (43). However, the interactions that occur between the strains that make up an infecting mycobiota is a topic yet to be explored.

### Conclusions.

Pollution is omnipresent; it greatly impacts living microbiota and contributes to the emergence of microbial pathogens (7, 18). Our previous studies with PCP highlighted that the soil mycobiota has a great capacity to degrade PCP but that there is a high price to pay in the form of increased pathogenic potential (21). It should be noted that many fungal taxa that exist in topsoil have a cosmopolitan distribution (44) and that soil is a common ecological niche where many opportunistic pathogenic fungi can be found (14, 44). This observation led us to consider new risks since (i) encounters between fungi and pollutants are inevitable, (ii) exposure of human airways to fungal spores is universal, (iii) the majority of the 500 fungal species that are able to cause diseases in humans have a primary environmental niche (45) but are opportunists (they do not appear to be specialized for colonization of a human host), and (iv) environmental fungi evolved pathogenicity-associated traits mostly in response to selection pressures outside the host—possibly linked to general stress adaptation (17), i.e., “accidental virulence” (46). These observations defined our working hypothesis: short-time (acute) exposure to a chlorinated aromatic pollutant raises the pathogenic potential of soil fungi. To test this hypothesis, we analyzed whether PCP and TCS (strong microbicidal agents that are frequently found in soil) potentiate the production of airborne fungal spores with a higher potential for virulence. Thus, we fabricated a cultivation system to allow for the collection of airborne spores (Fig. 1a), with spores being the principal agent through which human airways are expose to fungi; it is estimated that an adult inhales >100 A. fumigatus conidia per day (47). We noticed that the diversity of airborne fungal spores (Fig. 2d) is comparable to that observed previously for the same inoculum (pre- and postgrowth) (21) and is consistent with data from recent surveys of soil fungal communities (48). Airborne fungal spores comprise fungi that mostly belong to the wind dispersed, generalist Ascomycota phyla; with a clear dominance of A. fumigatus (90%), which is the most prevalent airborne fungal pathogen. It produces small conidia that allow for deep penetration of the pulmonary alveoli (20) and can cause disastrous aspergillosis infections in immunocompromised patients or in patients suffering from chronic obstructive pulmonary disease, asthma, or cystic fibrosis. Regardless of the fact that the inocula for all airborne spores had similar compositions, their relative *in vivo* virulence differed greatly. Spores produced under unpolluted conditions were systematically benign, while acute exposure of fungi to pollutants resulted in the production of airborne spores consisting of highly virulent strains. The continued presence of fungal (overall and cultivable) diversity (Fig. 2 and 3) inside living larvae, 72 h postinfection, revealed that the dominance of A. fumigatus was maintained. The most virulent strains isolated from larvae (Fig. 3) exhibited *in vivo* infection capacities close to that of an A. fumigatus clinical isolate (Fig. 4) but less than that of either test inoculum (Fig. 2). Since some strains could not be cultivated, the contribution of the less-abundant strains, comprising several that are rarely found to be pathogenetic, might be higher than anticipated. Another possibility is that synergisms occurring in the preparation of the nonaxenic spore are potentiators of the *in vivo* infection capacity. Consistent with this hypothesis, we observed that virulence increased when spores from two virulent strains were mixed prior to infection of the larvae (Fig. 4c). The taxonomic (Fig. 3) and functional parameters (see Table S6) analyzed here did not show any traits that could effectively differentiate virulent from avirulent strains. A. fumigatus strains have been isolated from environments containing hydrocarbons or phenols and are known to assimilate these substrates (49). It has been speculated that fungi’s ability to metabolize hydrocarbons may constitute a human virulence factor (50). We observed a high degree of heterogeneity in the capacities of strains to tolerate either pollutant and their susceptibility to two antifungal drugs (see Table S6 in the supplemental material). Neither tolerance nor susceptibility correlated with virulence potential. The A. fumigatus strains were split into two lineages (Fig. 3c), both containing avirulent and virulent strains. Variation in pathogenicity-associated traits is not restricted to species and lineages. Strains of A. fumigatus and other Aspergillus pathogens exhibit extensive genomic and phenotypic heterogeneity in their virulence and drug resistance profiles (51). Recently, it was shown that genetically identical conidia of different fungal species, including those of A. fumigatus, exhibit substantial phenotypic diversity (52). For example, *A fumigatus* strain-specific virulence is known to be modulated by nitrogen metabolism (40) (clearly dysregulated during PCP degradation [21]) and by the ability to thrive under low-oxygen conditions (39). The observed heterogeneity raises questions as to which genetic/epigenetic mechanisms contribute more to pathogenicity across human fungal pathogens and which are clinically relevant (53), but to date this remains largely unknown. Our study shows that soil pollution can promote an increase in the virulence of airborne spores of Aspergillus spp. compared to those released from an unpolluted source. It emphasizes pollution stress as being a possible driver of phenotypic adaptations that increase their pathogenicity, hence expanding the concept of an “environmental virulence training school” (54). A mechanistic understanding of the phenotypic strain plasticity resulting from exposure to pollution deserves a focused analysis in the future, with the aim being to reduce the emergence of more virulent and pathogenic fungi and to better understand the interplay between pollution and medical mycology.

## MATERIALS AND METHODS

### Study design.

We designed and three-dimensionally printed an incubation box—MC3000—able to maintain a constant airflow and accommodate membrane filters for the collection of airborne spores (Fig. 1a and b). Each incubation box housed 50 mL of minimal medium (21, 26) containing 1% glucose and 0.5% Phytagel, supplemented or not with pollutants at half-maximal effective concentrations (EC_50_) for each (10 mg L^−1^ and 50 mg L^−1^ for PCP and TCS, respectively) (21). The medium was inoculated with a soil mycobiota inoculum, and then boxes were sealed (Parafilm before placing the lid), followed by incubation for 10 days at 30°C (all cultures in triplicate, including negative controls [without any pollutant and abiotic controls]; two independent experiments were performed). During incubation, the circulated air ensured the dispersion of airborne spores toward the membrane filter. On day 10, the fungal spores were collected from the filters and further processed (see below). Spores deposited on to the media surface were also collected for validation experiments. The jellified medium, containing the mycelial matt, was also recovered and extracted to evaluate the decay of either pollutant by liquid chromatography (see the supplemental material). Airborne spores were used to study the community composition (amplicon sequencing) and to perform infection assays in G. mellonella. Pollutants potentially absorbed onto the airborne spores were extracted (see the supplemental material), and the ensuring extracts were tested individually (in triplicates). Complementary tests included analysis of the fungal diversity inside larvae and, subsequently, the isolation of cultivable strains and their characterization (viz. taxonomy, susceptibility to antifungals and to either pollutant and *in vivo* infection capacity).

### Soil mycobiota assays.

The mycobiota inoculum was recovered from soils (0- to 20-cm depth) sampled inside a cork oak forest in Tunisia, as previously reported (26). Each MC3000 cultivation system was inoculated with an inoculum corresponding to 0.5 g of soil. After incubation, the MC3000 filters were immersed in a saline solution (0.9%) containing 0.1% Tween20, to release the adherent spores. Spores on the surface of the cultures were independently collected. Spores were washed (18,000 × *g*, 20 min, 4°C), resuspended in a 30% glycerol solution, and stored at −80°C. A Neubauer chamber was used to count the number of spores (optical microscope, ×400 magnification), and their sizes were evaluated microscopically upon a calcofluor-white staining (Leica DM 6000B upright microscope, 63 × 1.4 NA oil immersion objective plus a 1.6× Optvar) through analysis of >20 independent microscopic fields per sample (ImageJ and XL-STAT, v1.8.0_172) as follows. The size bar was used to set scale, images were converted to 8-bit format, black-and-white thresholding was used to remove noise, potential holes in the particles were digitally filled, and the particle analysis command used. Results were trimmed (excluding areas <5 μm^2^) to remove remaining noise, registered, and further analyzed using XL-STAT (Addinsoft, v2014.5.03).

### Metataxonomics of fungal subpopulation.

The diversity of the collected airborne spores and of fungi subsisting inside larvae at 72 h postinfection were analyzed. The biomass of the spores or larvae (first frozen using liquid nitrogen and ground using a mortar and pestle) was disrupted, and the DNA was extracted. Afterward, the ITS2 region of fungal rDNA was amplified by PCR (55) (GeneAmp PCR system 2720; Applied Biosystems) using barcoded gITS7 and ITS4 primers (56). The obtained PCR products were sequenced on an Illumina MiSeq system, and the retrieved data were processed using the pipeline SEED 2.1 (57).

### *In vivo* infection tests.

The infection studies were performed as previously described using G. mellonella as the model organism (34). Injected larva (10^6^ spores [community], 10^7^ spores [axenic], and consortia of two strains matching their relative abundances in either test inoculum) or saline solution (pH 7.4), containing or not containing organic extracts obtained from airborne spores as a control, were examined daily for 96 or 120 h (dark, 37°C) and then scored daily according to the G. mellonella health index scoring system (58, 59). For each condition, 10 larvae were used, and at least three independent experiments were performed. Hemocyte levels were also counted in hemocytometer at 1, 4, 8, 12, and 24 h postinfection (extracted from three larvae per condition).

### Isolation of the cultivable fungal strains from the larvae (premortem).

Ground larval biomass in peptone water was spread onto solid malt extract agar (MEA) supplemented with 0.1% (vol/vol) chloramphenicol (in triplicate). Morphologically distinct fungal colonies were isolated by transfer to fresh media and cultivated for 4 days in MEA (>5 subculturing rounds); spores were processed as described above. DNA extraction, PCR conditions, primer sequences, and sequence assembly were similar to those described above (see “Metataxonomics of Fungal Subpopulation”). Genotyping of A. fumigatus isolates was performed by CD Genomics (Shirley, NY) using a panel of nine short tandem repeats as before (38). The MICs of antifungals and the EC_50_ levels of pollutants to each strain were determined using the EUCAST reference method (60) and on the basis of the hyphal radial growth rate, respectively. (Additional information regarding materials and methods is available in the supplemental material).

### Data availability.

See Data Set S1 in the supplemental material for detailed OTU tables obtained from amplicon sequencing data analysis and comparisons with previously acquired data. Data Set S2 presents the survival data for all infection experiments conducted with airborne fungal spores. Data Set S3 shows the Sanger sequencing results obtained for the fungal strains isolated from larvae. Data Set S4 includes the Kruskal-Wallis test results on the mean survival time of larvae upon infection with spores produced by each fungal strain.

The amplicon sequencing data have been deposited in the Sequence Read Archive (NCBI) under BioProject ID PRJNA900616.
