# Supplementary material for: Increased Production of Pathogenic, Airborne Fungal Spores upon Exposure of a Soil Mycobiota to Chlorinated Aromatic Hydrocarbon Pollutants
Source: Microbiol Spectr. 2023 Jun 7;11(4):e00667-23. doi: 10.1128/spectrum.00667-23 (PMC10434042; doi:10.1128/spectrum.00667-23)
Supplement: Supplemental file 1 — Supplemental material. Download spectrum.00667-23-s0001.docx, DOCX file, 1.3 MB [file spectrum.00667-23-s0001.docx]

**Increased production of pathogenic, airborne fungal spores upon exposure of a soil mycobiota to chlorinated aromatic hydrocarbon pollutants**

Celso Martins^aϕ^, Daryna Piontkivska^a#^, Dalila Mil-Homens^b,c#^, Paula Guedes^a,d^, João M.P. Jorge^a^, João Brinco^d^, Cátia Bárria^a^, Ariana C.F. Santos^a^, Ricardo Barras^a^, Cecília Arraiano^a^, Arsénio Fialho^b,c^, Gustavo H. Goldman^a,e^, Cristina Silva Pereira^a^

^a^Instituto de Tecnologia Química e Biológica António Xavier, Universidade Nova de Lisboa (ITQB NOVA), Av. da República, 2780-157, Oeiras, Portugal

^b^Institute for Bioengineering and Biosciences (iBB) and Institute for Health and Bioeconomy (i4HB), Instituto Superior Técnico, University of Lisbon, Av. Rovisco Pais, 1049-001 Lisboa, Portugal

^c^Department of Bioengineering, Instituto Superior Técnico, University of Lisbon, Av. Rovisco Pais, 1049-001 Lisboa, Portugal

^d^CENSE – Center for Environmental and Sustainability Research & CHANGE - Global Change and Sustainability Institute, NOVA School of Science and Technology, NOVA University Lisbon, Campus de Caparica, 2829-516 Caparica, Portugal

^e^Faculdade de Ciências Farmacêuticas de Ribeirão Preto, Universidade de São Paulo, Ribeirão Preto, Brazil

^ϕ^Present address: Center for Integrative Genomics, Faculty of Biology and Medicine, University of Lausanne, CH-1015 Lausanne, Switzerland

#*equal contributing authors*

^*^corresponding author: Cristina Silva Pereira (spereira@itqb.unl.pt)

**Full description of Materials and Methods**

**Chemicals.** Unless stated otherwise, all chemicals were purchased from Merck (Germany) at the highest purity grade available.

**MC3000.** The incubation boxes of the MC3000 system were built in polylactic acid printing filament, using an Anet A6 3D printer. Three distinct parts compose the incubation boxes: lid, collection box and filter adaptor (Figure 1a). An electronic circuit (Figure 1b) was built to operate the motors to spin fans at steady speed, ensuring air circulation inside the incubation box. The circuit is powered by a DC adapter (4.5 V) and regulated with zener diodes (3.3 V). Fusing resistors (2 Watts) were used to protect the motors (3 V). This circuit allowed for control of air turbulence, the intensity of which was maintained in all experiments.

**Soil mycobiota assays.** The *mycobiota inoculum* originated from soils sampled inside a cork oak forest in Tunisia (E008°'0051'00.00 N36°'0046'00.00) as previously described(1). The collected soil samples (0–20 cm depth) were pooled and sieved (<2 mm). To recover the mycobiota, a soil aliquot (15 g) was immersed (1:10, w/v) in a solution of 0.1% peptone (w/v) and 0.1% chloramphenicol (v/v) (60min, soft agitation, vacuum cycle every 20min), then sieved (pore sizes 500 µm, 210 µm then 100 µm)(1). 1 mL aliquots were stored at -80°C, each corresponding to 0.1 g of soil. For inoculation, the inoculum (ratio of 1:10, inoculum:medium) was mixed with the medium while warm (~40°C), then allowed to cool down.

Fungal spore collection: Following incubation, MC3000 filters were carefully removed, placed in 50 mL falcon tubes and fully immersed in saline solution (0.9%) containing 0.1% Tween 20, and sealed. To release the adherent spores, vigorous vortex cycles, followed by steps in an ultrasonic bath (3x, 10sec each) were used. Additionally, 20 mL of the same saline solution was added to the surface of the cultures, which were then gently scraped with a disposable cell scraper. Each spore suspension was filtered (glass wool) to remove the hyphal fragments, and centrifuged at 18000*g* (20min, 4 °C) to recover the spore pellet (washed twice with saline solution), then resuspended in a 30% glycerol solution and stored at ‑80 °C.

The number of spores in 10 µL aliquots were counted using a Neubauer chamber with the aid of a standard optical microscope at 400x magnification. Aliquots of 10 µL were stained with calcofluor-white; dilutions were performed whenever necessary to improve cellular separation, and were visualised using a Leica DM 6000B upright microscope equipped with an Andor iXon 885 EMCCD camera and controlled with MetaMorph V5.8 software, using the 63x 1.4 NA oil immersion objective plus a 1.6x optvar. At least 20 independent microscopic fields of each sample were captured. Images were analysed with ImageJ (version 1.8.0_172) as follows: the size bar was used to set scale; images were converted to 8-bit format; black and white thresholding was used to remove noise; potential holes in the particles were digitally filled and the particle analysis command used. Results were trimmed (excluding areas <5 µm^2^) to remove remaining noise, registered and further analysed using XL-STAT (Addinsoft, version 2014.5.03).

**Half maximal Effective Concentration (EC_50_)** **of each pollutant against the soil mycobiota.**

The EC_50_ of PCP and TCS were determined using 5-mL cultures (6-well plates; 2 plates *per* replicate). Growth media (1% w/v of glucose in a mineral minimal media(2), MMG) containing either 19, 38, 95, 190, 380 or 760 µM of PCP, or 35, 87.5, 175, 350, 500 or 1000 µM of TCS were mixed with the soil mycobiota inoculum (ratio of 1:10, inoculum:medium), incubated at 30ºC, 90 rpm for seven days (triplicates, including negative controls - unpolluted). After incubation, 50 µL from each biological replicate (group of twelve wells) were spread over MEA and the numbers of colony forming units (CFUs) monitored daily for 5 days and compared to that of the negative controls (triplicates). The EC_50_ value for PCP was defined and previously published by us for the community under study - 38 µM (10 mg·L^-1^)(2), whereas the EC_50_ of TCS was herein determined - 172 µM (50 mg·L^-1^).

**Chemical extractions and chromatographic analyses.** To evaluate the degradation of either pollutant after 10 days of incubation, the jellified media (with fungal mycelia or without in the abiotic controls) were freeze dried and ground using a pestle and mortar. The ground media were then extracted with HPLC grade methanol using an ultrasonic bath for 30min. The samples were centrifuged at 13,000*g* for 15min at 4 °C and the supernatant recovered. This step was repeated twice for each sample. The organic extracts were dried under gentle airflow and stored until further analysis. To evaluate the existence of pollutants adsorbed to the airborne spores, samples were extracted with HPLC grade methanol using 3 cycles of 10min in an ultrasonic bath interchanged with 5min of bead beating using a TissueLyzer LT Adapter (Qiagen, Germany) at maximum speed (1 g of glass beads *per* sample, equal amounts of beads of 0.5 and 0.1 mm). The organic extracts were dried under gentle airflow and stored until further analysis.

Analyses of these extracts were performed using high performance liquid chromatography (HPLC) with a diode array detector (DAD) and fluorescence detector (FLD). HPLC analysis was performed on a LC system equipped with a Quaternary Pump (G7111B) and a vial sampler (G7129A) (Infinity II, 1260 Series, Agilent Technologies, USA), coupled to a diode array detector (G1315B) and a fluorescence detector (G1321A) (Agilent 1100 Series). The UV wavelength was set to scan 200 to 500 nm. The system was operated with LC OpenLab software (version 2.15.26). The methanolic extracts were dried and resuspended in 100 µL of methanol. Analytes separation was performed using a CORTEC T3 2.7 μm column (4.6 x 100 mm; Waters) and an Onyx SecurityGuard C18 cartridges (5 x 4.6 mm; Phenomenex). The oven was set to 36 °C. HPLC runs were performed at a constant flow of 1.5 ml·min^-1^, in gradient mode. The eluents used were acetonitrile/Mili-Q water solutions (solution A: 5/95; solution B: 95/5), with 0.1% phosphoric acid. All eluents were filtered through Nylon 66 membranes (pore size 0.45 μm; Bellefonte). The gradient run was set to 1min 30% B, after 60% B until 5min, then 95% B until 10min, and until 97% B on min 11 where it was held constant for 1min and then 30% B until 13min. Post-run equilibrium was carried out for 2min. The reference standards for the target compounds and extract samples were analysed at 212 nm for PCP and 282 for TCS. LD and LQ were, respectively, 130 and 390 µg.L^-1^ for PCP and 80 and 240 µg.L^-1^ for TCS.

***In vivo* infection tests**. *Galleria mellonella* larvae were reared on a pollen grain and bee wax diet at 25 °C in the dark and used at a final developmental stage with a weight of 225 ± 25 mg. The infection studies in *G. mellonella* were performed as previously described(3). Each larva was injected with 10^6^ spores (community) or 10^7^ spores (axenic and consortia of two strains: 1:1 for a mix of two *A. fumigatus* strains, and 10:1 for a mix of *A. fumigatus* with *A. niger*, to match their relative abundances in the either Test-inoculum). A set of 10 larvae was used for each condition and a micrometre was adapted to control the volume of a microsyringe and inject 5 µl of 'spores' suspension into each larva via the hindmost left proleg, previously sanitised with 70% (v/v) ethanol. Following injection, the larvae were placed in Petri dishes, stored (dark, 37 °C) and examined daily for 96h or 120h. Larvae were considered dead when they displayed no movement in response to touch. Each larva was also scored daily according to the *G. mellonella* Health Index scoring system(4) that covers four parameters: larvae activity, cocoon formation, melanisation, and survival. The larvae health status scores are >9 (to a maximum of 10) for a healthy larva and < 9 for an infected larva. The control larvae were injected with a saline solution (pH 7.4) containing or not the organic extracts obtained from spores (*see above*). For each condition 10 larvae were used and at least three independent experiments were performed. For testing the infection capacity of the spores generated by each fungal isolate (*see below*), the spores were harvested from fresh cultures following at least 5 rounds of sub-culturing (in standard media, *i.e.* not supplemented with either pollutant). To analyse haemocytes levels, hemolymph was extracted from 3 larvae of each condition at 1, 4, 8, 12 and 24h post-infection. The larvae were punctured in the abdomen with a sterile needle and the outflowing lymph was immediately transferred to a microtube containing a few crystals of phenylthiourea to prevent melanisation. A 10-fold dilution was performed with saline solution and hemocytes density was determined by enumeration using a hemocytometer. A minimum of three independent assays were performed for all experiments.

**Metataxonomics of each fungal sub-population.** DNA extraction. The biomass of the spores or larvae was disrupted by performing 3 consecutive cycles of heating (5 min, 90 ° C) followed by beating with glass beads (1 g of glass beads *per* sample, equal amounts of beads of 0.5 and 0.1 mm) using a TissueLyzer LT Adapter (Qiagen, Germany) for 5min at maximum speed, with the aid of an extraction buffer (50 mM NaH_2_PO_4_, 50 mM NaCl, 500 mM Tris-HCl, 5% SDS, pH 8; 600 µL *per* culture). Afterwards, the sample was mixed with a half volume of each: phenol and chloroform containing isoamyl alcohol (24:1; hereafter defined as solution A); shaken for 2min and centrifuged (5min, 2,400*g*) to recover the upper supernatant that was re-extracted with an equal volume of solution A, and recovered as described before. To this mixture 1/3 volume of 6M NaCl and 1/10 volume of 10% of cetyl trimethylammonium bromide (CTAB) in 0.7M of NaCl were added, and the mixture was incubated for 30min at 65 ºC. After cooling to room temperature, an equal volume of solution A was added, shaken and centrifuged (2 min, 1,400*g*) to recover the supernatant. Finally, DNA was precipitated in 2/3 volume of isopropanol and 1/10 volume of acetate solution (3M) during 20min at room temperature, and recovered by centrifugation (20min, 6,800*g*). The DNA pellet was washed with 200 µL of ethanol (70%), recovered by centrifugation as before, air dried for 60min, eluted in 50 µL of TE buffer (Qiagen, Germany) and finally stored at -20 ºC. Prior to use, the DNA samples were cleaned using the GeneClean Turbo kit for 100-300 kb fragments (MP Biomedicals, USA) following the manufacturer instructions. Illumina Sequencing. The ITS2 region of fungal rDNA was amplified by PCR in a GeneAmp PCR system 2720 (Applied Biosystems) using barcoded gITS7 and ITS4 primers (gITS7, '5'- GTG ART CAT CGA RTC TTT G-'3'; ITS4, '5'- TCC TCC GCT TAT TGA TAT GC-'3') (5) in technical triplicate, including quality controls, as previously described (6). The quality of the PCR products was monitored by gel electrophoresis. The technical replicates were pooled and sequenced on an Illumina MiSeq system operated by the Gene Expression Unit at the Instituto Gulbenkian de Ciência (Oeiras, Portugal).

Amplicon sequencing data analysis. The amplicon sequencing data were processed using the pipeline SEED 2.1 (7) as applied before. Brieﬂy, paired-end reads were joined using FASTQ-join (8). The ITS2 region was extracted using ITSx1.0.11(9) before processing. The chimeras were identified using USEARCH 8.1.1861 and deleted. Sequences were clustered using UPARSE implemented within USEARCH(10) at a 97% similarity level. The most abundant sequences were selected for each cluster, and the closest hits were identiﬁed using BLASTn against GenBank. Singletons were discarded.

**Isolation of the cultivable fungal strains from the larvae (pre-mortem).** Ground larval biomass was resuspended in peptone water (1:10 larvae biomass:peptone water, 0.1% w/v, incubated for 1h, 37 °C, 100 rpm) and then spread onto solid Malt Extract Agar (MEA) supplemented with 0.1% (v/v) chloramphenicol to inhibit bacterial growth (in triplicate). Morphologically distinct fungal colonies (checked daily) were isolated by transfer to fresh media; isolates were cultivated for 4 days in MEA (spores were harvested and stored as described above). DNA extraction was performed using the Quick-DNA^TM^ Fungal/Bacterial Microprep Kit (Zymo Research). All strains were characterised by ITS sequencing (primers gITS7 and ITS4). Details of the PCR conditions, primer sequences, and sequence assembly were similar to those described above (Metataxonomics). Sequence similarity searches were performed in public databases (GenBank;http://www.ncbi.nlm.nih.gov/) with BLAST (version 2.5.0).

**Microsatellite genotyping.** Genotyping was performed by CD Genomics (NY, USA) with a panel of nine short tandem repeats (TRs) as previously described(11). In brief, three separate multiplex PCRs amplifications were performed to obtain three dinucleotide, three trinucleotide and three tetranucleotide loci fragments. Each PCR mixture contained 1x reaction buffer, 0.3 mM of the corresponding amplification primers, 0.2 mM deoxynucleotide triphosphates, 0.5 U of Hot Start Taq DNA Polymerase and 1 ng of genomic DNA. Amplification was performed using the following thermal protocol: 5min of initial denaturation at 95 °C, followed by 35 cycles of 30sec of denaturation at 95 °C, 30sec of annealing at 60 °C and 30sec of extension at 72 °C, with final extension for 30min at 60 °C. The obtained fragments were denatured at 95 °C for 3min in a mixture containing 1.0 μl of PCR product, molecular weight internal standard and 0.05% formamide. The fragment analysis was performed on the Applied Biosystems 3730xl DNA Analyzer, and the sizes determined using GeneMapper Software 5.

**Minimal Inhibitory Concentrations (MICs) of antifungals and EC_50_ levels of pollutants against each fungal strain.** MICs were determined using a microbroth dilution format according to the EUCAST reference method (12). Tests were carried out with RPMI-1640 medium (R6504, with L-glutamine and without sodium bicarbonate) supplemented with 2% glucose and 0.165 mol·L^-1^ of 3- (N-morpholino) propanesulfonic acid (MOPS) and a pH adjusted to 7.0. The concentrations tested, all in mg·L^-1^, ranged from 256 to 0.5 for amphotericin B, from 8 to 0.016 for posaconazole, and from 512 to 1 for either pollutant. Spore suspensions were prepared to a final working inoculum of 10^6^ conidia·mL^-1^. Growth and negative controls were included in all tests. The microplates were incubated at 37 ºC for 48h. The lowest concentration that showed no growth under microscopic observation was considered the MIC. EC_50_ levels of pollutants were assessed on basis of hyphal radial growth rate of filamentous fungi. Petri plates (55 mm) were prepared to contain 10 mL of mineral minimal media jellified with 1% phytagel and supplemented with PCP (final concentrations: 1, 2, 2.5, 4, 5, 7.5, 10, 25, 50 and 100 mg·L^-1^) or TCS (final concentrations: 1, 2, 4, 5, 7.5, 10, 25, 50, 100, 150 and 300 mg·L^-1^). Controls, without pollutants, were also made. The assay was carried out by inoculating 1 µl of spore suspension containing 2×10^5^ conidia into the centre of the plate and incubated at 37 °C for 48h. Radial growth of mycelia (colony diameter, mm) was measured using a Vernier calliper (associated error ± 0.05 mm), plates that did not displayed visible growth were visualised under the microscope to confirm growth inhibition.

**Statistical analyses.** The boxplots showing the dispersion and containing each measured spore were generated using ggplot2 package in R (4.2.1), and differences between conditions were tested using pair-wise Student’s t-test, Levene’s test and F-test. The survival curves were computed using survminer package and the plots generated using the ggsurvplot function included in the package ggplot2 in R (4.2.1), statistical differences were computed using the log rank test embedded in survminer. The mean survival time of the larvae upon inoculation with spores from each axenic isolate were assessed by computing 10000 Monte Carlo simulations followed by Kruskal-Wallis test using the software XL-STAT (Addinsoft, version 2014.5.03).

The calculations to obtain the EC_50_ values of PCP and TCS towards the mycobiota or each fungal strain, were computed by adjusting measurements to a logistic regression using the dose effect tool of XL-STAT (Addinsoft, version 2014.5.03). The percentage of growth inhibition for each tested concentration (IC) was calculated using the Gompertz model available at the dose effect tool of XL-STAT (Addinsoft, version 2014.5.03).

**Degradation of the pollutants during cultivation using the MC3000 cultivation system**

**Table S1.** Pollutants' levels detected at the end of the MC3000 experiments (mg·L^-1^). Decay levels were calculated relative to the Abiotic Control of each pollutant – medium spiked with the pollutant which underwent incubation in the MC3000 boxes under the same conditions as the remaining biotic assays. The limits of detection (LD) and quantification (LQ) were, respectively, 130 and 390 µg·L^-1^ for PCP and 80 and 240 µg·L^-1^ for TCS.

| **Samples** | **mg·L^-1^** | | **Decay (%)** | **Average decay (%)** | **Std dev.** |
| --- | --- | --- | --- | --- | --- |
| PCP Abiotic Control | 11.64 |  | |  |  |
| PCP | 3.18 | 72.67 | | 70.80 | 3.65 |
| PCP | 3.89 | 66.59 | |  |  |
| PCP | 3.13 | 73.14 | |  |  |
| TCS Abiotic Control | 58.89 |  | |  |  |
| TCS | 3.36 | 94.29 | | 94.89 | 2.26 |
| TCS | 4.13 | 93.00 | |  |  |
| TCS | 1.54 | 97.39 | |  |  |

**
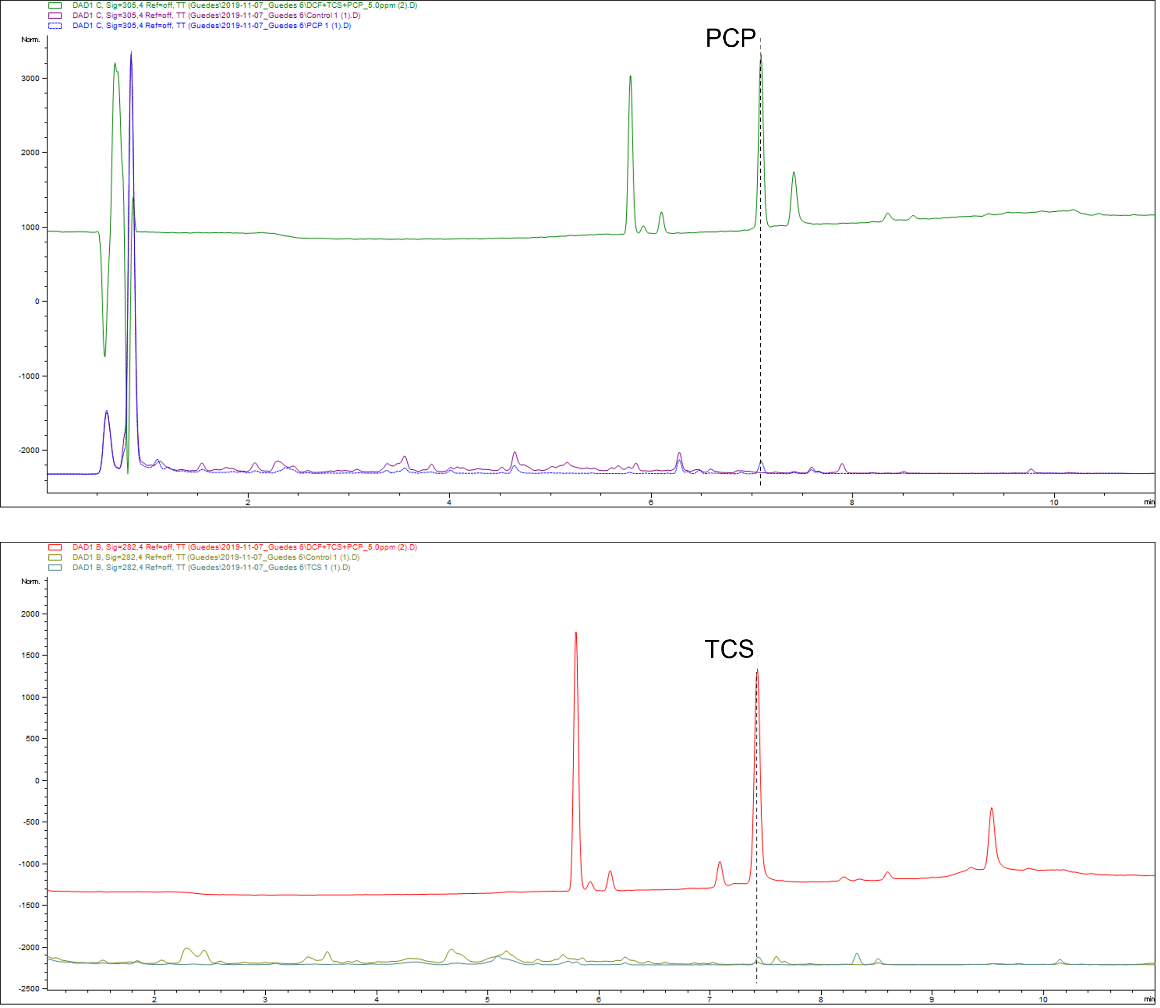
**

**Figure S1 –** HPLC chromatograms of the MC3000 media collected after incubation (below), compared with a standard containing 5 ppm of either PCP or TCS (top).

**Table S2 – Statistical tests comparing the airborne spores’ sizes collected from different conditions using the MC3000 system**

| Comparison\Test | Fisher | Levene | t-test |
| --- | --- | --- | --- |
| PCP vs Control | **< 0.0001** | **< 0.0001** | **< 0.0001** |
| TCS vs Control | **0.0007** | **< 0.0001** | **< 0.0001** |
| TCS vs PCP | **< 0.0001** | **0.0002** | **0.0001** |

**Preliminary comparison between the sizes of airborne and surface spores collected upon MC3000 cultivation**


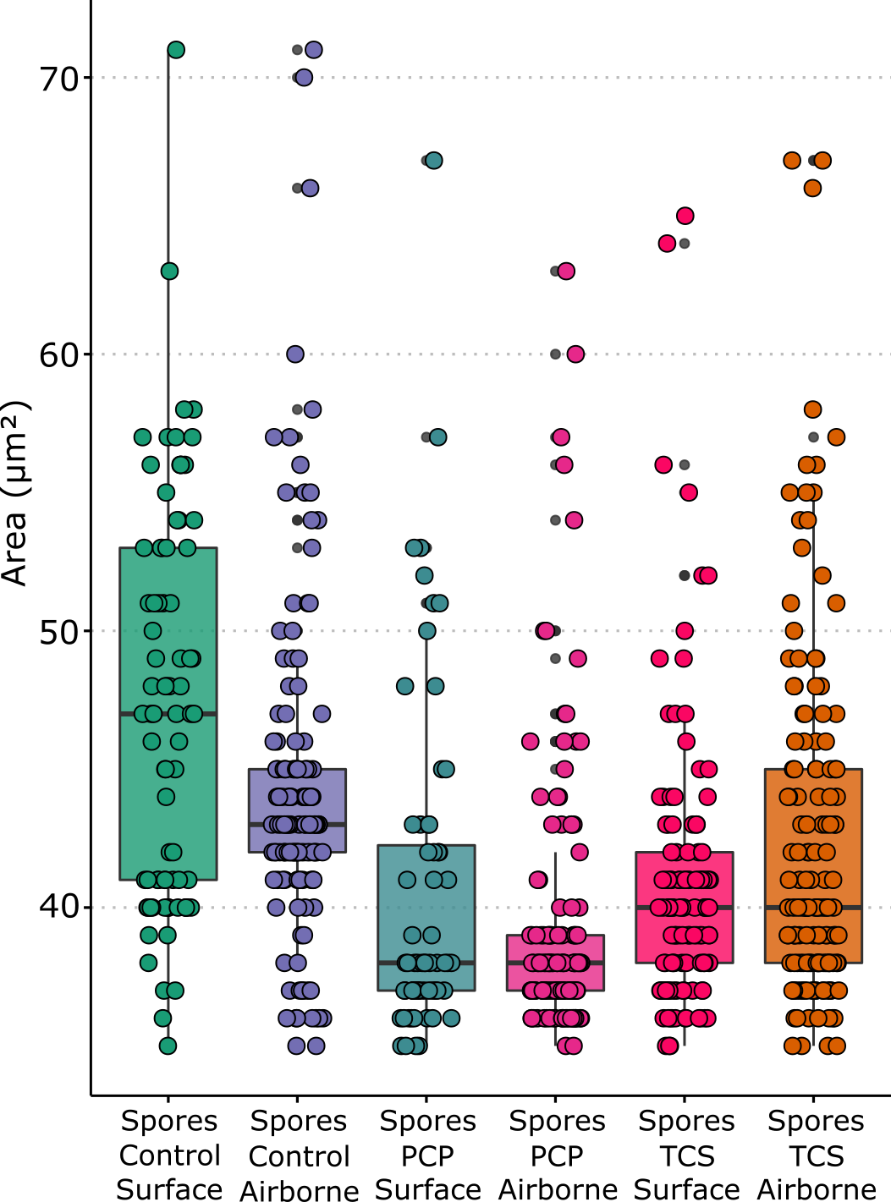


**Figure S2 –** Boxplots of preliminary measurements of spores' sizes (area in µm^2^) of both fractions collected upon MC3000 experiments: airborne and deposited on the media surface. Overall, the average size of the spores produced under test conditions (PCP and TCS) was smaller than those collected in unpolluted conditions (control) both for airborne and surface spore fractions.

**The 33 species clusters identified by metataxonomics analysis of the airborne fungal spores collected upon MC3000 cultivation**

**Table S3 –** Species clusters obtained using metataxonomics. For the OTUs matching strains isolated from larvae prior to their infection with either inoculum, the individual isolates' sequences were aligned, and clusters discriminated whenever possible. For the remaining species the accession numbers were considered to cluster OTUs in species groups.

|  | **Airborne spores** | | | **Inside larvae** | | |
| --- | --- | --- | --- | --- | --- | --- |
| **Species cluster** | **Unpolluted** | **PCP** | **TCS** | **Unpolluted-inoculum** | **PCP-inoculum** | **TCS-inoculum** |
| *Aspergillus fumigatus* strains: Cluster 1 or 3 | 92385 | 95372 | 93324 | 98791 | 85084 | 88106 |
| *Aspergillus niger* strains: Cluster 2 | 4608 | 3511 | 5256 | 861 | 10783 | 11543 |
| *Trichoderma scalesiae* | 327 | 150 | 211 | 229 | 3878 | 34 |
| *Trichosporon coremiiforme* | 1035 | 326 | 359 | 5 | 8 | 8 |
| *Sporidiobolus salmonicolor* | 474 | 145 | 177 | 1 | 1 | 1 |
| *Amorphotheca resinae* | 364 | 166 | 155 | 5 | 1 | 1 |
| *Aspergillus fumigatus* strain AEM007 | 302 | 132 | 186 | 22 | 62 | 49 |
| *Pyronemataceae* sp. | 161 | 72 | 103 | 2 | 2 | 150 |
| *Syncephalastrum racemosum* strains: Cluster 5, 6 or 7 | 114 | 37 | 63 | 54 | 26 | 32 |
| *Trichoderma saturnisporum* | 122 | 28 | 42 | 0 | 1 | 1 |
| *Rhizopus arrhizus* | 24 | 9 | 13 | 13 | 5 | 5 |
| *Fusarium oxysporum* | 19 | 11 | 27 | 7 | 8 | 7 |
| *Cladosporium flabelliforme* | 14 | 9 | 3 | 1 | 62 | 3 |
| *Vanrija albida* | 13 | 7 | 18 | 7 | 4 | 7 |
| *Alternaria alternata* | 6 | 2 | 5 | 1 | 53 | 0 |
| *Phlebiopsis* sp. | 6 | 3 | 7 | 1 | 6 | 17 |
| *Pleosporales* sp. | 6 | 2 | 12 | 5 | 2 | 4 |
| *Trichoderma* sp. | 8 | 4 | 3 | 1 | 4 | 9 |
| *Alternaria dactylidicola* | 1 | 1 | 1 | 0 | 12 | 0 |
| *Trichoderma virens* | 1 | 3 | 8 | 2 | 0 | 1 |
| *Mucor fragilis* | 6 | 3 | 6 | 0 | 0 | 0 |
| *Apiotrichum vadense* | 2 | 0 | 3 | 0 | 0 | 3 |
| *Solicoccozyma phenolica* | 3 | 0 | 1 | 1 | 1 | 2 |
| *Ascosphaera major* | 3 | 1 | 1 | 0 | 1 | 2 |
| *Preussia persica* | 1 | 1 | 1 | 0 | 1 | 1 |
| *Malassezia restricta* | 1 | 0 | 2 | 1 | 0 | 1 |
| *Wallemia peruviensis* | 1 | 1 | 1 | 0 | 1 | 1 |
| *Stachybotrys chartarum* | 0 | 0 | 1 | 0 | 3 | 1 |
| *Pyronema domesticum* | 0 | 1 | 1 | 1 | 1 | 0 |
| *Meira nashicola* | 0 | 1 | 1 | 0 | 0 | 1 |
| *Chaetomium afropilosum* | 0 | 1 | 0 | 0 | 1 | 1 |
| *Parmelia sulcata* | 1 | 1 | 1 | 1 | 0 | 1 |
| *Umbelopsis dimorpha* | 1 | 0 | 1 | 1 | 1 | 1 |

**Exploratory infection experiment comprising pooled fractions of airborne and surface spores collected upon MC3000 cultivation**

We performed infection experiments containing both fractions of spores (airborne and surface collected) pooled together as inoculum (10^7^ spores *per* larva).

**Table S4 –** Survival rates of *Galleria mellonella* larvae after 24h of infection with pooled spore’s fractions (airborne and surface) collected upon MC3000 experiments.

|  | **Survival rates 24h (%)** |
| --- | --- |
| **Blank** | 100 |
| **Spores Control** | 90 |
| **Spores PCP** | 10 |
| **Spores TCS** | 0 |

The experiment was stopped after 24h because almost all larvae inoculated with Test-inocula were dead. This result, together with the comparable sizes of airborne and deposited spores (Table S2), confirms the suitability of the airborne spore sub-population to monitor potential risks of exposure through inhalation.

**The organic extracts obtained from the airborne fungal spores collected in polluted conditions do not contain traces of adsorbed pollutants**

The airborne fungal spores collected upon the cultivation of the soil mycobiota under polluted conditions using the MC3000 system were extracted and analysed as specified above. No traces of pollutants nor degradation products were detected in the extracts (30-fodl concentrated), as seen in Figure S3.

**
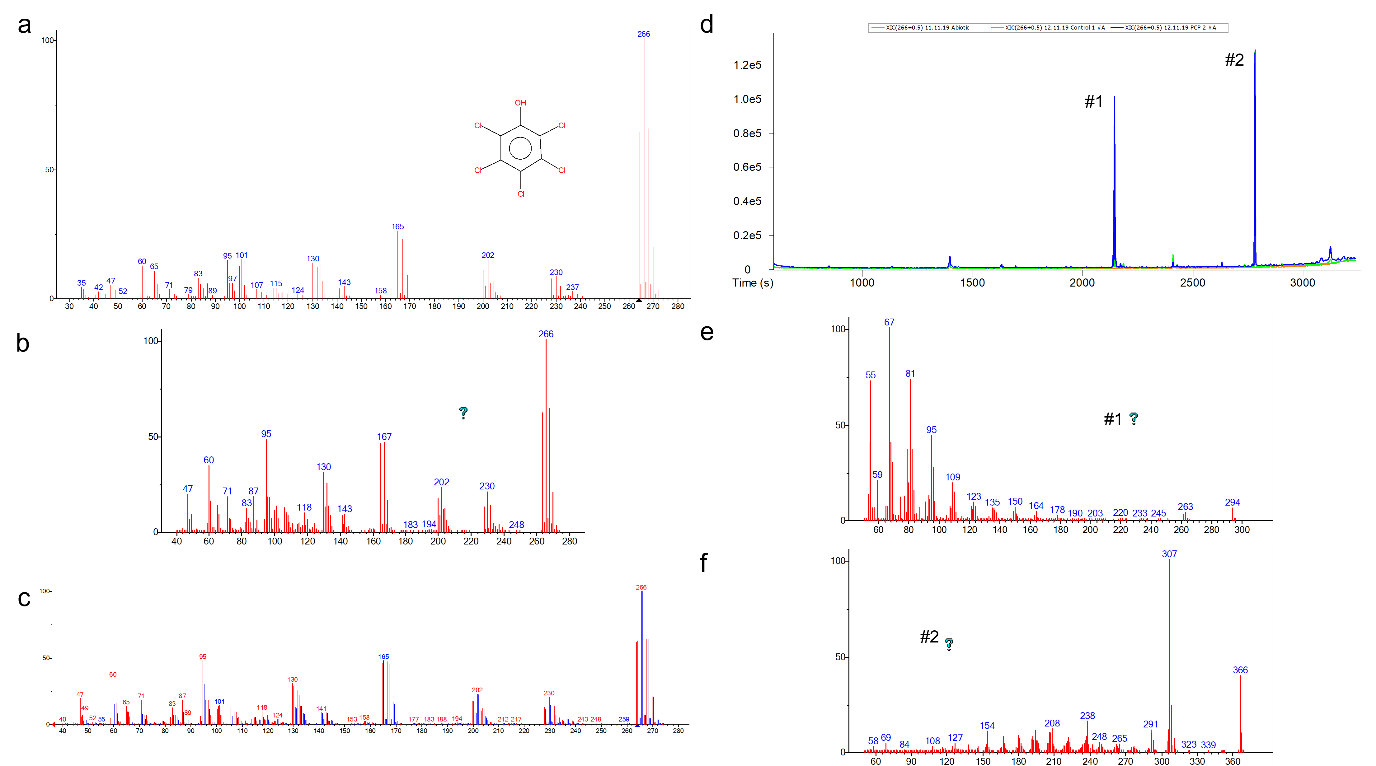
**

**Figure S3 –** The database (NIST) profile of PCP is displayed in a), which served as comparison with the b) chemical standard showing a clear c) ionization pattern overlap, as expected. In the d) XIC profile of the spores’ extract (30x concentrated) two major peaks were detected, however the ionization patterns of neither e) the peak #1 nor f) the peak 2 corresponded to PCP. No traces of PCP could be detected in the high concentrated extract of the spores collected under PCP conditions.

**
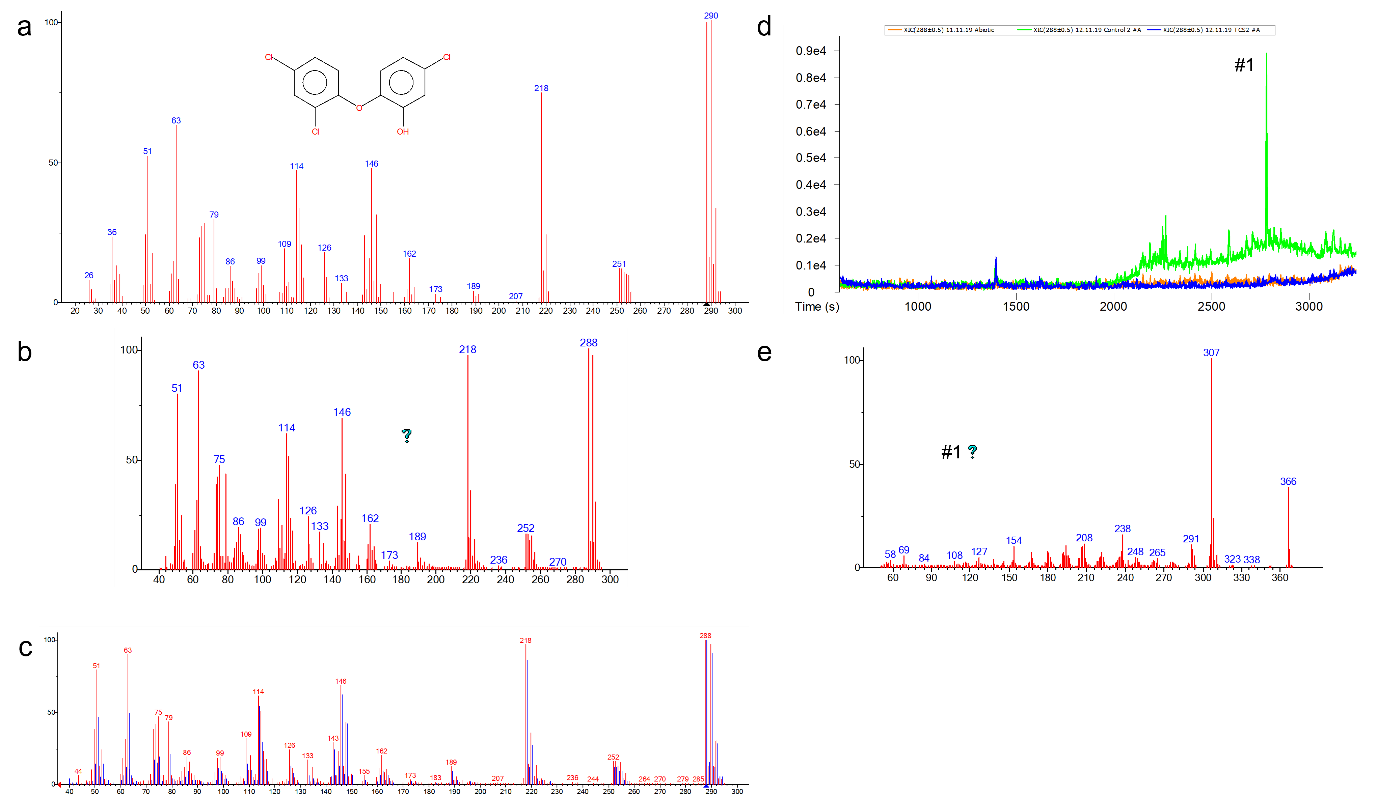
**

**Figure S4 –** The database (NIST) profile of TCS is displayed in a), which served as comparison with the b) chemical standard showing a clear c) ionization pattern overlap, as expected. In the d) XIC profile of the spores’ extract (30x concentrated) one major peak was detected, however the ionization patterns of e) this peak (#1) did not match with TCS. No traces of TCS could be detected in the high concentrated extract of the spores collected under TCS conditions.

**Expression of *G. mellonella* antimicrobial peptides encoding genes**

To assess the expression levels of antimicrobial peptides as response to infection with the airborne fungal spores collected at all conditions (tests and control), we performed *q*RT-PCR of the regions encoding gallerimycin, inducible metalloproteinase inhibitor (IMPI), lysozyme and galliomycin as described above.

RNA extraction*.* For the extraction of RNA from *G. mellonella*, sets of 20 larvae were infected as previously described for *in vivo* infection(13). At 1, 4, 8, 12, and 24h after injection, three living larvae *per* set were cryopreserved, sliced, and homogenised in 1 mL of TRIzol reagent (Sigma-Aldrich, St. Louis, MO, USA). Whole-animal RNA was extracted according to the 'manufacturer's protocol. After extraction, RNA was treated with an RNase-free DNase set (Qiagen, Germantown, MD, USA). The purified RNA was quantified spectrophotometrically (NanoDrop ND-1000, Wilmington, DE, USA). RNA integrity was evaluated by gel electrophoresis and RNA concentration was estimated using a Nanodrop 1000 machine (Nanodrop Technologies,Wilmington, DE, USA).

Quantitative Real-Time PCR*.* Reverse transcription coupled with quantitative PCR (RT-qPCR) was performed with a Real Time Thermal Cycler qTower system (Analytik Jena, Jena, Germany) using a SensiFast SYBR kit (Bioline, London, UK) according to the 'supplier's instructions. cDNA was synthesised from 1 µg of purified RNA with the SensiFast cDNA synthesis kit (Bioline, London, UK). The pair of primers used are gallerimycin (5´-CGCAATATCATTGGCCTTCT-3' and 5´-CCTGCAGTTAGCAATGCAC-3'); IMPI (5'- AGATGGCTATGCAAGGGATG-3'and 5´-AGGACCTGTGCAGCATTTCT-3'); lysozyme (5'-TCCCAACTCTTGACCGACGA-3'and 5´-AGTGGTTGCGCCATCCATAC-3'); galliomycin (5´-TCGTATCGTCACCGCAAAATG-3' and 5'- GCCGCAATGACCACCTTTATA-3') and actin (5´-ATCCTCACCCTGAAGTACCC-3' and 5´-CCACACGCAGCTCATTGTA-3'). The expression of *G. mellonella* genes was normalised to the amount of the housekeeping gene actin. Relative quantification of gene expression was calculated by using the DDCt (Ct is threshold cycle) method(14).


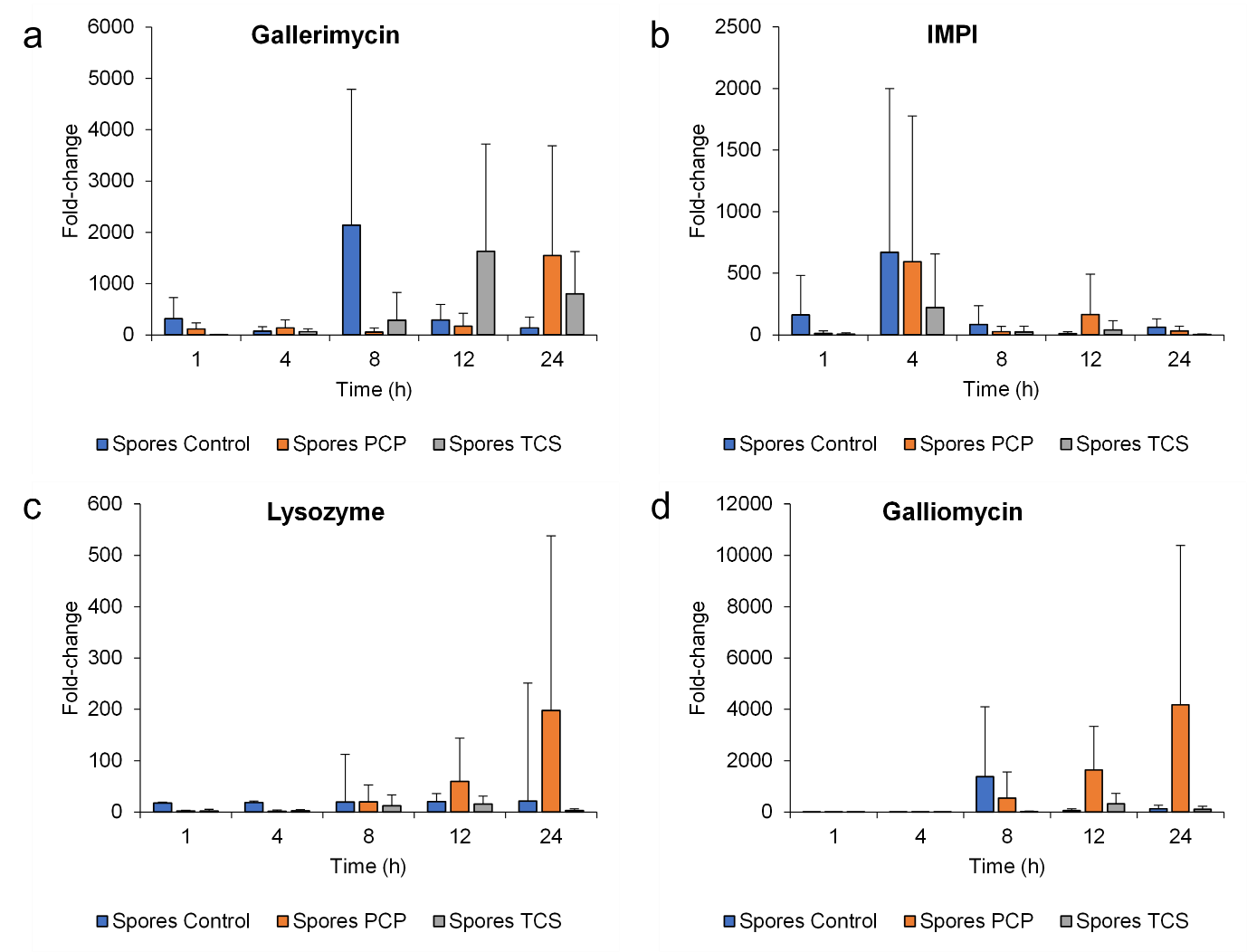


**Figure S5 –** *q*RT-PCR expression (fold-change relatively to actin housekeeping gene) of the antimicrobial peptides Gallerimycin (a), Inducible metalloproteinase inhibitor (IMPI, b), Lysozyme (c) and Gallomycin (d) measured in *Galleria mellonella* larvae during the first 24h of infection with airborne spores of all conditions (Unpolluted (control), PCP and TCS).

The levels of antimicrobial peptides produced by the larvae showed substantial variability (not unusual though(15)) hence failing to reveal statistical sound differences between the different Test- and Control- conditions.

**Microsatellite genotyping of *Aspergillus fumigatus* isolates**

**Table S5 –** Microsatellite genotyping of the *A. fumigatus* strains isolated from the *G. mellonella* larvae upon infection studies.

|  | STRAf2A (GA) | | STRAf2B (AG) | | STRAf2C (CA) | |
| --- | --- | --- | --- | --- | --- | --- |
| Sample ID | Fragment size | Repeat no. | Fragment size | Repeat no. | Fragment size | Repeat no. |
| AEM004 | 184.6 | 21 | 164.6 | 30 | 154.8 | 8 |
| AEM006 | 177.1 | 17 | 160.8 | 28 | 154.9 | 8 |
| AEM007 | 184.7 | 21 | 164.5 | 30 | 154.9 | 8 |
| AEM008 | 184.7 | 21 | 164.5 | 30 | 154.8 | 8 |
| AEM009 | 184.7 | 21 | 164.6 | 30 | 154.9 | 8 |
| AEM012 | 177.2 | 17 | 160.9 | 28 | 154.9 | 8 |
| AEM015 | 177.1 | 17 | 160.9 | 28 | 154.9 | 8 |
| Af293 | 192.5 | 25 | 140.1 | 17 | 173.3 | 17 |
| Clinical isolate | 178.9 | 18 | 154.7 | 25 | 173.3 | 17 |
|  |  |  |  |  |  |  |
|  | STRAf3A (TCT) | | STRAf3B (AAG) | | STRAf3C (TAG) | |
| Sample ID | Fragment size | Repeat no. | Fragment size | Repeat no. | Fragment size | Repeat no. |
| AEM004 | 145.7 | 12 | 159.8 | 9 | 84.4 | 7 |
| AEM006 | 193.9 | 28 | 160.1 | 9 | 120.8 | 19 |
| AEM007 | 146.0 | 12 | 160.2 | 9 | 84.3 | 7 |
| AEM008 | 145.8 | 12 | 160.2 | 9 | 84.3 | 7 |
| AEM009 | 145.5 | 12 | 162.1 | 9 | 84.4 | 7 |
| AEM012 | 194.3 | 28 | 160.2 | 9 | 120.8 | 19 |
| AEM015 | 194.2 | 28 | 159.8 | 9 | 120.8 | 19 |
| Af293 | 246.3 | 45 | 193.2 | 20 | 132.9 | 23 |
| Clinical isolate | 206.3 | 32 | 167.3 | 11 | 120.8 | 19 |
|  |  |  |  |  |  |  |
|  | STRAf4A (TTCT) | | STRAf4B (CTAT) | | STRAf4C (ATGT) | |
| Sample ID | Fragment size | Repeat no. | Fragment size | Repeat no. | Fragment size | Repeat no. |
| AEM004 | 198.6 | 12 | 180.0 | 8 | 184.0 | 10 |
| AEM006 | 177.9 | 7 | 175.9 | 7 | 164.9 | 5 |
| AEM007 | 198.7 | 12 | 180.2 | 8 | 184.0 | 10 |
| AEM008 | 198.3 | 12 | 180.2 | 8 | 184.1 | 10 |
| AEM009 | 198.7 | 12 | 180.2 | 8 | 184.2 | 10 |
| AEM012 | 178.2 | 7 | 176.0 | 7 | 164.7 | 5 |
| AEM015 | 178.0 | 7 | 175.9 | 7 | 165.0 | 5 |
| Af293 | 190.1 | 10 | 184.0 | 9 | 176.3 | 8 |
| Clinical isolate | 190.0 | 10 | 187.9 | 10 | 176.2 | 8 |

**Minimal Inhibitory Concentrations (MIC) of antifungals and Half Maximal Effective Concentration levels (EC_50_) of the pollutants towards each fungal strain**

The complete set of data regarding IC_50_ and MIC values of antifungals and pollutants, respectively, is available in the tables below.

**Table S6 –** IC_50_ of pollutants and MICs of antifungals towards each fungal strain isolated from *Galleria mellonella* larvae upon infection with airborne spores collected at all tested conditions (Unpolluted (Control), PCP and TCS).

|  |  | **EC_50_ (mg·L^-1^)** | | **MIC (mg·L^-1^)** | |
| --- | --- | --- | --- | --- | --- |
| **Isolate** | **Species** | **PCP** | **TCS** | **Amphotericin B** | **Posaconazole** |
| AEM004 | *A. fumigatus* | 10.540 | 36.913 | 8.000 | 0.125 |
| AEM005 | *S. racemosum* | 8.111 | 21.429 | 2.000 | 2.000 |
| AEM006 | *A. fumigatus* | 6.637 | 26.834 | 8.000 | 0.250 |
| AEM007 | *A. fumigatus* | 8.152 | 23.023 | 64.000 | 0.063 |
| AEM008 | *A. fumigatus* | 6.865 | 20.973 | 128.000 | 0.250 |
| AEM009 | *A. fumigatus* | 8.552 | 22.124 | 128.000 | 0.250 |
| AEM010 | *S. racemosum* | 4.372 | 10.406 | 1.000 | 2.000 |
| AEM011 | *A. niger* | 5.695 | 18.416 | 1.000 | 0.500 |
| AEM012 | *A. fumigatus* | 9.622 | 18.839 | 8.000 | 0.125 |
| AEM013 | *S. racemosum* | 6.453 | 12.576 | 4.000 | 4.000 |
| AEM014 | *A. niger* | 5.752 | 16.141 | 2.000 | 0.500 |
| AEM015 | *A. fumigatus* | 10.397 | 16.431 | 4.000 | 0.125 |
| AF18  (clinical isolate) | *A. fumigatus* | 8.048 | 4.298 | 4.000 | 0.250 |

**Supplementary References**

1. Varela A, Martins C, Núñez O, Martins I, Houbraken JA, Martins TM, Leitão MC, McLellan I, Vetter W, Galceran MT, Samson RA, Hursthouse A, SIlva Pereira C. 2015. Understanding fungal functional biodiversity during the mitigation of environmentally dispersed pentachlorophenol in cork oak forest soils. Environmental microbiology 17:2922-2934.

2. Martins C, Varela A, Leclercq CC, Núñez O, Větrovský T, Renaut J, Baldrian P, Silva Pereira C. 2018. Specialisation events of fungal metacommunities exposed to a persistent organic pollutant are suggestive of augmented pathogenic potential. Microbiome 6:208.

3. Durieux M-F, Melloul É, Jemel S, Roisin L, Dardé M-L, Guillot J, Dannaoui É, Botterel F. 2021. *Galleria mellonella* as a screening tool to study virulence factors of *Aspergillus fumigatus*. Virulence 12:818-834.

4. Tsai CJ-Y, Loh JMS, Proft T. 2016. *Galleria mellonella* infection models for the study of bacterial diseases and for antimicrobial drug testing. Virulence 7:214-229.

5. Ihrmark K, Bödeker I, Cruz-Martinez K, Friberg H, Kubartova A, Schenck J, Strid Y, Stenlid J, Brandström-Durling M, Clemmensen KE. 2012. New primers to amplify the fungal ITS2 region–evaluation by 454-sequencing of artificial and natural communities. FEMS microbiology ecology 82:666-677.

6. Žifčáková L, Větrovský T, Howe A, Baldrian P. 2016. Microbial activity in forest soil reflects the changes in ecosystem properties between summer and winter. Environmental microbiology 18:288-301.

7. Větrovský T, Baldrian P, Morais D. 2018. SEED 2: a user-friendly platform for amplicon high-throughput sequencing data analyses. Bioinformatics.

8. Aronesty E. 2013. Comparison of sequencing utility programs. The open bioinformatics journal 7.

9. Nilsson RH, Veldre V, Hartmann M, Unterseher M, Amend A, Bergsten J, Kristiansson E, Ryberg M, Jumpponen A, Abarenkov K. 2010. An open source software package for automated extraction of ITS1 and ITS2 from fungal ITS sequences for use in high-throughput community assays and molecular ecology. fungal ecology 3:284-287.

10. Edgar RC. 2013. UPARSE: highly accurate OTU sequences from microbial amplicon reads. Nature methods 10:996-998.

11. de Valk HA, Meis JF, Curfs IM, Muehlethaler K, Mouton JW, Klaassen CH. 2005. Use of a novel panel of nine short tandem repeats for exact and high-resolution fingerprinting of *Aspergillus fumigatus* isolates. Journal of clinical microbiology 43:4112-4120.

12. Testing SoASTotEECfAS. 2008. EUCAST technical note on the method for the determination of broth dilution minimum inhibitory concentrations of antifungal agents for conidia-forming moulds. Clinical microbiology and infection 14:982-984.

13. Bárria C, Mil-Homens D, Pinto SN, Fialho AM, Arraiano CM, Domingues S. 2022. RNase R, a new virulence determinant of Streptococcus pneumoniae. Microorganisms 10:317.

14. Livak KJ, Schmittgen TD. 2001. Analysis of relative gene expression data using real-time quantitative PCR and the 2− ΔΔCT method. methods 25:402-408.

15. Mak P, Zdybicka-Barabas A, Cytryńska M. 2010. A different repertoire of Galleria mellonella antimicrobial peptides in larvae challenged with bacteria and fungi. Developmental & Comparative Immunology 34:1129-1136.
